# Recovery from 6-month spaceflight at the International Space Station: muscle-related stress into a proinflammatory setting

**DOI:** 10.1096/fj.201801625R

**Published:** 2019-01-08

**Authors:** Miriam Capri, Cristina Morsiani, Aurelia Santoro, Manuela Moriggi, Maria Conte, Morena Martucci, Elena Bellavista, Cristina Fabbri, Enrico Giampieri, Kirsten Albracht, Martin Flück, Severin Ruoss, Lorenza Brocca, Monica Canepari, Emanuela Longa, Irene Di Giulio, Roberto Bottinelli, Paolo Cerretelli, Stefano Salvioli, Cecilia Gelfi, Claudio Franceschi, Marco Narici, Jörn Rittweger

**Affiliations:** * Department of Experimental, Diagnostic, and Specialty Medicine, University of Bologna, Bologna, Italy;; † Galvani Interdepartmental Center, University of Bologna, Bologna, Italy;; ‡ National Research Council–Institute of Molecular Bioimaging and Physiology (CNR–IBFM), Segrate, Milan, Italy;; § Italian National Olympic Committee (CONI), Rome, Italy;; ¶ Department of Physics and Astronomy, University of Bologna, Bologna, Italy;; ‖ Faculty of Medical Engineering and Technomathematics, FH Aachen University of Applied Sciences, Aachen, Germany;; # Institute of Biomechanics and Orthopaedics, German Sport University, Cologne, Germany;; ** Department of Orthopaedics, University of Zürich, Zürich, Switzerland;; †† Department of Molecular Medicine, University of Pavia, Pavia, Italy;; ‡‡ Sport Medicine Center, University of Pavia, Pavia, Italy;; §§ Centre of Human and Applied Physiological Sciences, King’s College London, London, United Kingdom;; ¶¶ Fondazione Salvatore Maugeri, Institute of Hospitalization and Scientific Care (IRCCS), Scientific Institute of Pavia, Pavia, Italy;; ‖‖ Department of Biomedical Sciences for Health, University of Milan, Milan, Italy;; ## IRCCS, Istituto Ortopedico Galeazzi, Milan, Italy;; *** Department of Applied Mathematics, Institute of Information Technology, Mathematics, and Mechanics (ITMM), Lobachevsky State University of Nizhny Novgorod–National Research University (UNN), Nizhny Novogoro, Russia;; ††† Department of Biomedical Sciences, University of Padova, Padua, Italy;; ‡‡‡ Institute of Aerospace Medicine, German Aerospace Center (DLR), Cologne, Germany; and; §§§ Department of Pediatrics and Adolescent Medicine, University of Cologne, Cologne, Germany

**Keywords:** microRNA-206, inflamma-miRs, proteasome, SERPINA1

## Abstract

The Sarcolab pilot study of 2 crewmembers, investigated before and after a 6-mo International Space Station mission, has demonstrated the substantial muscle wasting and weakness, along with disruption of muscle’s oxidative metabolism. The present work aimed at evaluating the pro/anti-inflammatory status in the same 2 crewmembers (A, B). Blood circulating (c-)microRNAs (miRs), c-proteasome, c-mitochondrial DNA, and cytokines were assessed by real-time quantitative PCR or ELISA tests. Time series analysis was performed (*i.e.*, before flight and after landing) at 1 and 15 d of recovery (R+1 and R+15, respectively). C-biomarkers were compared with an age-matched control population and with 2-dimensional proteomic analysis of the 2 crewmembers’ muscle biopsies. Striking differences were observed between the 2 crewmembers at R+1, in terms of inflamma-miRs (c-miRs-21-5p, -126-3p, and -146a-5p), muscle specific (myo)-miR-206, c-proteasome, and IL-6/leptin, thus making the 2 astronauts dissimilar to each other. Final recovery levels of c-proteasome, c-inflamma-miRs, and c-myo-miR-206 were not reverted to the baseline values in crewmember A. In both crewmembers, myo-miR-206 changed significantly after recovery. Muscle biopsy of astronaut A showed an impressive 80% increase of α-1-antitrypsin, a target of miR-126-3p. These results point to a strong stress response induced by spaceflight involving muscle tissue and the proinflammatory setting, where inflamma-miRs and myo-miR-206 mediate the systemic recovery phase after landing.—Capri, M., Morsiani, C., Santoro, A., Moriggi, M., Conte, M., Martucci, M., Bellavista, E., Fabbri, C., Giampieri, E., Albracht, K., Flück, M., Ruoss, S., Brocca, L., Canepari, M., Longa, E., Di Giulio, I., Bottinelli, R., Cerretelli, P., Salvioli, S., Gelfi, C., Franceschi, C., Narici, M., Rittweger, J. Recovery from 6-month spaceflight at the International Space Station: muscle-related stress into a proinflammatory setting.

It is known that short- and long-term spaceflights are associated with physiologic and biologic changes of the human body (1–3). Currently, long-term orbiting flights are regularly performed to serve the International Space Station (ISS) missions, and deep space missions (*e.g.*, to the moon or Mars) are thought to be feasible soon (4). Among the many bodily effects, those related to the skeletal-muscle apparatus and brain appear to be particularly relevant in terms of possible health risks and difficulty to revert the changes after landing (5). Many of the space-related changes are detrimental to the body, and it has been suggested that microgravity could be seen as a model of ageing (6).

Access to astronauts is quite limited, which is a considerable impediment to the generation of knowledge in space medicine. Luckily, the possibility of measuring advanced blood biomarkers, such as microRNAs (miRs), and pro- and anti-inflammatory cytokines offer the intriguing opportunity of easily monitoring crew health concerning the physiologic and stress-associated challenges of spaceflight. In addition, circulating (c-)markers are promising tools for the evaluation of healthy and unhealthy ageing trajectories (7). Thus, blood is an informative tissue in which the presence and the concentration of markers may indicate not only tissue/organ injuries or suffering status but also epigenetic changes that may propagate in all the body, especially in the case of c-miRs. In fact, many of these molecules are able to modulate inflammatory signaling pathways, in particular the inflamma-miRs (miR-21-5p, -126-3p, -146a-5p), which were found to be increased or dysregulated in the blood with ageing or pathologic conditions (8).

The Sarcolab pilot study has studied the neuromuscular adaptations to long-term space flight in 2 crewmembers before and after a 6-mo ISS mission and has demonstrated substantial muscle wasting and weakness, along with disruption of muscle’s oxidative metabolism, as a result of spaceflight (9). The muscle atrophy observed with spaceflight has some analogy with the age-associated loss of muscle mass (sarcopenia) (10). In both conditions, the loss of muscle mass could contribute to the increase of c-markers networking with the stress response and proinflammatory status as well as inflammageing along with life span (11–13). Further support for such a view is provided by the recent observation that body core temperature is increased in space in a way that is independent of impeded heat dissipation and which seems to be linked with an inflammatory response (14).

The driving hypothesis is that spaceflight, as a prolonged stressor, and recovery may favor a proinflammatory status, increasing the molecular “garbage,” such as misplaced molecules (15), which in turn may favor the inflammatory stress conditions. To this purpose, the present work attempts to evaluate the pro- and anti-inflammatory status in the 2 crewmembers (A and B) who spent ∼6 mo in space. Blood c-miRs, c-proteasome, c-mitochondrial DNA (mtDNA), and cytokines were evaluated before flight and after 1 and 15 d of recovery and correlated with muscle proteomic analysis. All data were acquired taking into account the main question: How similar are the 2 crewmembers’ responses to spaceflight and recovery after 1 and 15 d from landing?

## MATERIALS AND METHODS

### Subjects and time series sampling

Two crewmembers of the same sex and similar age, A and B, were tested before and after a 6-mo ISS mission. Ethics committee approval was obtained in accordance with the ethical standards presented in the Declaration of Helsinki and its later amendments. Accordingly, informed consent was obtained prior to study inclusion and information on in-flight countermeasure training was obtained *via* data sharing with the National Aeronautics and Space Administration as previously reported (9). Blood/plasma and soleus muscle tissue samples were obtained between 76–79 d before flight (preflight) from both astronauts and at 24 h and 15 d after return (R+1 and R+15, respectively), in accordance with the previous work (9). Crewmembers’ data were compared to a healthy and age-matched control group recruited in Bologna, Italy. Blood and biopsy samples were obtained in the morning, after having food withheld overnight, both in astronauts as well as in the control group. The control group never underwent spaceflight. In particular, 19 plasma samples were collected from 6 healthy volunteers at 4 different times (up to 7 mo, but some samples were not obtained). Plasma samples were processed and frozen within 2 h after blood drawing. A large set of c-molecules, including miRs, proteasome, mtDNA, and cytokines as described in **Table 1**, was assessed in both crewmembers and control group. In particular, for each measurement, time series data of control group have been combined as baseline reference, thus including intraindividual and seasonal variability.

**TABLE 1:** List of blood c-markers assessed in the current work and related references

Marker	Biologic endpoint	Reference
miR-206	Myo-miR, skeletal muscle	53
miR-133a-3p	Myo-miR, skeletal muscle	53
miR-21-5p	Inflamma-miR, proinflammatory and pro-osteogenesis	8, 40, 54
miR-126-3p	Inflamma-miR, proinflammatory, expressed in endothelial cells	8, 50
miR-146a-5p	Inflamma-miR, proinflammatory and procell senescence	8, 55
miR-122-5p	Liver integrity and function	56, 57
miR-145-5p	Cell proliferation and tumor suppressor	58, 59
miR-363-3p	Cell growth and differentiation	60
c-proteasome	Tissue injury, pathologic condition	16
IL-6	Systemic proinflammatory citokine	61
Leptin	Adipokine involved in metabolism	12
TGF-β1	Anti-inflammatory cytokine	62
mtDNA	Proinflammatory	63, 46

### C-proteasome quantification

C-proteasome analysis was performed in plasma by a self-developed ELISA assay, as previously described (16). Briefly, ELISA plates were coated with a mouse monoclonal antibody toward 20S proteasome-subunit α6 (Enzo Life Sciences, Farmingdale, NY, USA), and 20S purified proteasome in a concentration range of 0–100 ng/ml was used as calibration standard. An antiproteasome rabbit pAb (obtained from an expert research team) and then a peroxidase-conjugated mouse anti-rabbit IgG (Jackson ImmunoResearch Laboratories, West Grove, PA, USA) were applied for antigen detection. OD-values were determined at 450 nm. Every sample was tested in triplicate, and the mean of the values was reported.

### C-miRs relative quantification

Total RNA was extracted from plasma-EDTA samples (100 µl) with a Total RNA Purification Kit (Norgen Biotek, Thorold, ON, Canada) according to the manufacturer’s protocol. In addition, 20 fmol of spike-in cel-miR-39 (Qiagen, Venlo, The Netherlands) was added to the plasma samples at the lysis step as control for RNA extraction efficiency. Eight miRs were chosen, having a crucial and referenced regulatory role (see Table 1), and were measured by quantitative RT-PCR in plasma samples: miR-21-5p, -126-3p, -146a-5p, -145-5p, -133a-3p, -206, -122-5p, and -363-3p. These miRs were measured by applying TaqMan technologies (Thermo Fisher Scientific, Waltham, MA, USA); this method consists of an miR-specific retrotranscription, in which RNA is first transcribed in cDNA for each miR, then cDNA is used as a template for the quantitative PCR reaction. MiR relative expression was calculated by Δ*C*_*t*_ method using 2 replicates for each measurement. *C*_t_ values were normalized with miR-16-5p after validation of its stability along the time series analysis (17).

### C-mtDNA relative quantification

Total DNA was isolated from plasma-EDTA samples using Quick-gDNA MiniPrep Kit (Zymo Research, Irvine, CA, USA). To quantify the free mtDNA copy number, a real-time quantitative PCR SYBR Green assay was performed using a standard curve as calibration. Assays were performed in duplicate by Rotor-Gene Q 6000 Detector (Qiagen), using SYBR GreenER Mix (Thermo Fisher Scientific) and forward/reverse primer (specific for 69-bp fragment internal to the ND1 mt-gene fragment used for calibration). Specificity of PCR products was confirmed by melting curve analysis. Each run was repeated 3 times. Standard curve was set up using 10-log serial dilution of stock solution containing from 10^−8^ to 10^−4^ mtDNA copies/μl. To determine mtDNA copies, a 217-bp fragment, corresponding to *MT-ND1* gene, was amplified by PCR and loaded on 1% agarose gel. DNA corresponding to the 217-bp band was isolated and quantified by absorbance and used as a calibrator. mtDNA copy number of calibrator was obtained by the total DNA concentration divided by amplicon weight. The latter was estimated as follows: (217 bp × MWt)/A, where MWt denotes the MW of double-stranded DNA (6.6 × 10^5^ g/mole), and A denotes Avogadro’s number (6.02 × 10^23^ molecules/mole).

### Cytokines/leptin quantification

IL-6, TGF-β1 and Leptin concentration were measured in plasma samples with commercial ELISA kit (R&D Systems, Minneapolis, MN, USA) according to the manufacturer’s instructions. All measurements were performed in duplicate, and the average values were used in the statistical analyses.

### Muscle proteins

Protein extraction and minimal labeling with cyanine dyes (Cy3 and Cy5), and 2-dimensional (2-D) separation and analyses were performed as previously described (9). Proteins of interest were identified by peptide mass fingerprinting (PMF) utilizing a matrix-assisted laser desorption/ionization time-of-flight mass spectrometer (Ultraflex III-MALDI ToF/ToF Mass Spectrometer; Bruker, Billerica, MA, USA), as previously described (18). In particular, a search was carried out by correlation of uninterpreted spectra to Mammalia entries in the National Center for Biotechnology Information (Bethesda, MD, USA) database (ID:20090430; 8,483,808 sequences; 2,914,572,939 residues). When this approach was unsuccessful additional searches were performed using electrospray ionization–tandem mass spectrometry (MS/MS), as previously described (19). For further information about PMF and liquid chromatography–MS/MS, data are listed in **Tables 2** and **3**. A representative example of heat shock protein β-1 (HSPB1) analysis with matrix-assisted laser desorption/ionization–ToF PMF and electrospray ionization–MS/MS is reported in supporting information (Supplemental Fig. S4). Proteomic analyses were performed in triplicates.

**TABLE 2:** The entire list of proteins differentially expressed in all comparisons for both crewmembers (A and B), together with statistical analyses (values reported as P in columns in reference to Tukey’s test), protein Ac number and gene name.

Protein	Accession no.	Gene	Subject A				Subject B			
			R+1 *vs.* preflight		R+15 *vs.* preflight		R+1 *vs.* preflight		R+15 *vs.* preflight	
			Tukey’s test	% fold change	Tukey’s test	% fold change	Tukey's test	% fold change	Tukey’s test	% fold change
Heat shock protein beta-1	P04792	*HSPB1*	7.28E−04	−31			1.58E−04	29	9.47E−04	19
Heat shock protein beta-1	P04792	*HSPB1*	1.66E−03	−22	7.09E−03	−18	1.55E−03	30	3.93E−03	24
60 kDa heat shock protein, mitochondrial	P10809	*HSPD1*			5.03E−03	−22				
Heat shock–related 70 kDa protein 2	P54652	*HSPA2*			9.15E−03	49				
Endoplasmic reticulum chaperone BiP	P11021	*HSPA5*							6.99E−03	15
Annexin A2	P07355	*ANXA2*					2.13E−03	−27	4.44E−04	30
α-1-antitrypsin*^a^*	P01009	*SERPINA1*	4.33E−03	66	3.93E−03	81			7.06E−03	23
Peroxiredoxin-2	P32119	*PRDX2*	4.32E−03	21			1.61E−03	29		
Peroxiredoxin-6	P30041	*PRDX6*	8.91E−03	−17						
Superoxide dismutase	Q7Z7M6	*SOD2*	9.41E−03	−19						
Catalase	P04040	*CAT*	8.83E−03	23						
Catalase	P04040	*CAT*	4.12E−03	50						
Glutathione *S*-transferase Mu 2	P28161	*GSTM2*			3.93E−03	43				
Protein/nucleic acid deglycase DJ-1	Q99497	*PARK7*					2.57E−03	35		
Tripartite motif-containing protein 72	Q6ZMU5	*TRIM72*	5.33E−03	−25	3.93E−03	−26	4.05E−03	−31		

a SERPINA1; protein identified by liquid chromatography–MS/MS.

**TABLE 3:** The entire list of proteins differentially expressed in reference to protein Ac number, gene name, theoretical molecular mass, isoelectric points, and MS data

Protein	Accession no.	Gene	MW (kDa)	pI	Matched/searched peptides	Protein mascot score	Sequence coverage (%)	MS/MS sequence	MS/MS score	*m*/*z*	*z*	Range (aa)
Heat shock protein beta-1	P04792	*HSPB1*	22.3	9.1	8/17	116	32.2	LFDQAFGLPR	63	1163.635	1	28–37
Heat shock protein beta-1	P04792	*HSPB1*	22.3	9.1	9/23	126	44.7	LFDQAFGLPR	88	1163.633	1	28–37
60 kDa heat shock protein, mitochondrial	P10809	*HSPD1*	61.2	5.6	13/15	161	26.5	AAVEEGIVLGGGCALLR	95.6	1684.89	1	430–446
Heat shock–related 70 kDa protein 2	P54652	*HSPA2*	69.9	5.5	15	143	25.8	TTPSYVAFTDTER	104	1487.726	1	38–50
Endoplasmic reticulum chaperone BiP	P11021	*HSPA5*	72.1	4.9	10/18	114	20.4	EFFNGKEPSR	38	1210.6	1	376–385
Annexin A2	P07355	*ANXA2*	38.6	8.5	14/33	174	36.0	QDIAFAYQR	42.5	1111.53	1	69–77
α-1-antitrypsin*^a^*	P01009	*SERPINA1*	46.7	5.3	5	257	12.7	SPLFMGK	28	398.297	2	405–411
								AVLTIDEK	30	444.823	2	360–367
								SVLGQLGITK	70	508.404	2	325–334
								LSITGTYDLK	69	555.873	2	315–324
								VFSNGADLSGVTEEAPLK	30	917.528	2	335–352
Peroxiredoxin-2	P32119	*PRDX2*	21.9	5.6	9/13	158.0	44.4	QITVNDLPVGR	66.6	1211.66	1	139–149
Peroxiredoxin-6	P30041	*PRDX6*	25.0	6.0	12/22	180.0	49.1	LPFPIIDDR	77.9	1085.594	1	96–106
Superoxide dismutase	Q7Z7M6	*SOD2*	22.2	7.0	7/19	99.0	38.4	AIWNVINWENVTER	48.8	1743.882	1	179–192
Catalase	P04040	*CAT*	59.7	7.0	10/36	75	23.9	AFYVNVLNEEQR	59	1481.746	1	445–456
Catalase	P04040	*CAT*	59.7	7.0	11/37	102.0	30.0	AFYVNVLNEEQR	85.3	1481.746	1	445–456
Glutathione *S*-transferase Mu 2	P28161	*GSTM2*	25.7	6.0	10/38	103.0	45.0	DCGATWVVLGHSER	87	1586.727	1	85–98
Protein/nucleic acid deglycase DJ-1	Q99497	*PARK7*	19.9	6.4	8/12	105.0	39.2	GAEEMETVIPVDVMR	77.2	1675.811	1	13–27
Tripartite motif-containing protein 72	Q6ZMU5	*TRIM72*	52.6	6.0	9/18	103.0	16.4	LLPAAEAHAR	38	1048.59	1	119–128

Ac, ascesion number; pI, isoelectric points.

a Protein identified by liquid chromatography–MS/MS.

### Bioinformatic analysis

Validated miR targets were identified by means of Dianatools (mirPath v.3.0) using TarBase v.7.0 for the union of inflamma-miRs targets and apart, the validated targets of muscle-specific (myo)-miR-206. Kyoto Encyclopedia of Genes and Genomes (KEGG) pathways and Gene Ontology (GO) category analyses were applied to identify the most significant molecular network involving the transcripts regulated by the selected miRs.

### Statistical analysis and modeling

Data obtained by astronauts were compared with the control distribution, and a *z* score test was applied for each value. Values of *P* < 0.05 were considered significant. Data exploiting was obtained by considering each molecule as an independent variable and was standardized by means of a box-cox transform. The different ranking of each variable was obtained by graphing the *z* score of each molecular target and comparing it with the baseline population. Proteomic statistical analysis was performed using the DeCyder 1.0 extended data analysis module. Protein filters were set to select only those protein spots that matched >90% of the gel images, and these protein spots were included in data analysis. Statistically significant differences of 2-D–difference gel electrophoresis data were computed by paired 1-way ANOVA (2-sided) coupled to Tukey’s test; the significance level was set at α <0.01. In addition, the false discovery rate was applied as a multiple testing correction method to keep the overall error rate as low as possible (20). Two independent analyses were performed for crewmembers A and B by comparing R+1 *vs.* preflight and R+15 *vs.* preflight for each member.

## RESULTS

**Figure 1:**
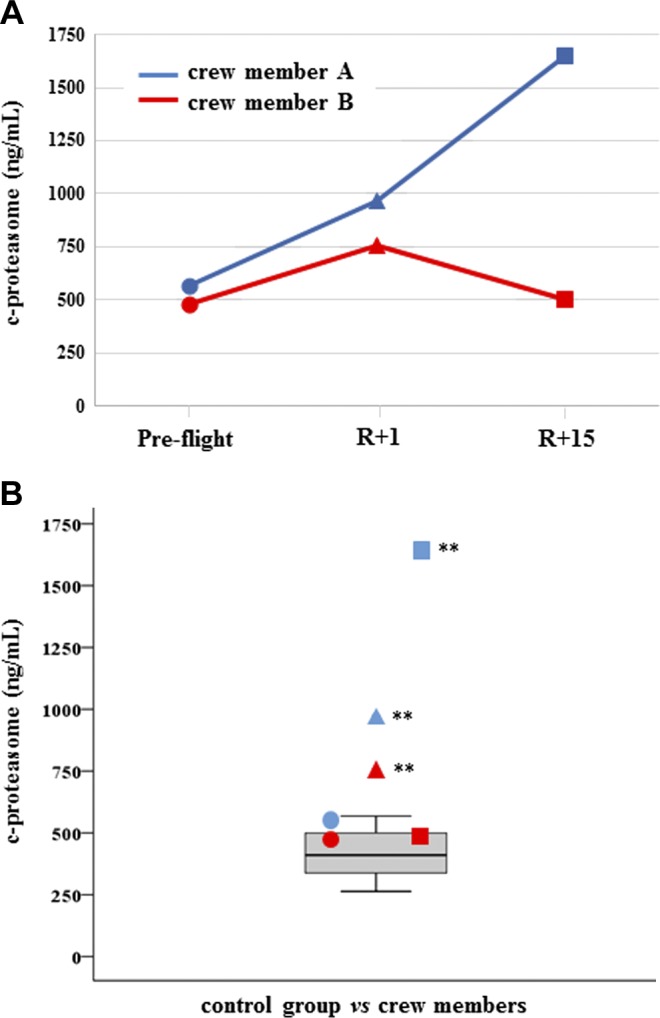
C-proteasome. *A*) Measurements are reported in dependence of time in both crewmembers. Circles represent preflight, triangles (R+1 d) landing time, and squares denote (R+15 d) recovery time. *B*) C-proteasome values of crewmembers are compared with age-matched control distribution (19 measurements). Blue: crewmember A; red crewmember B. **P ≤ 0.01 (*z*-score test).

**Figure 2:**
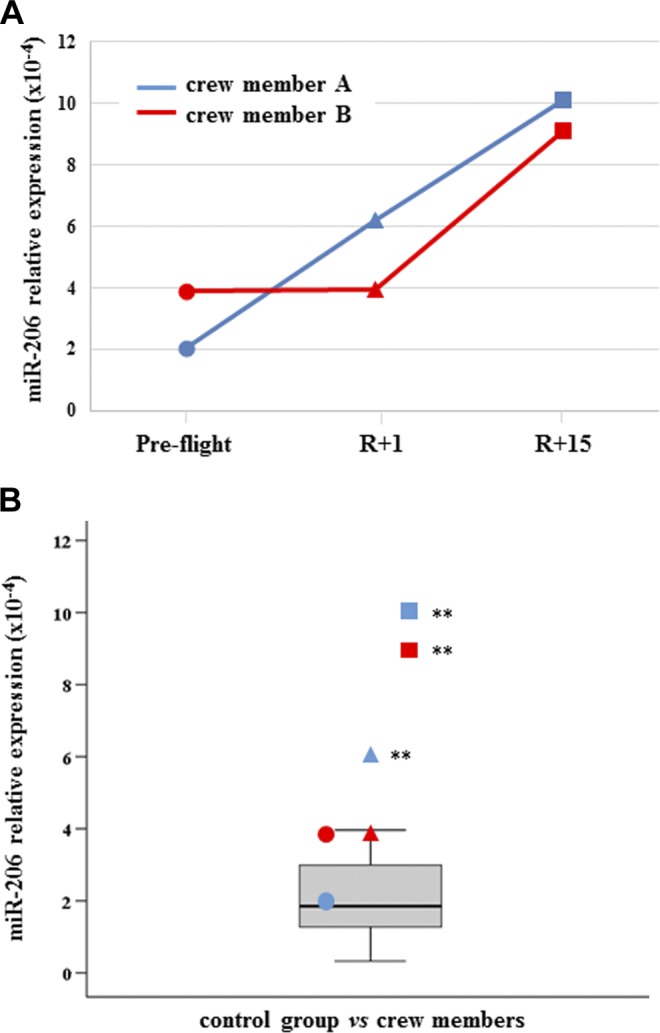
C-myo-miR-206. *A*) Measurements are reported in dependence of time in both crewmembers. For explanation of symbols see Fig. 1. *B*) C-myo-miRs-206 values of crewmembers are compared with age-matched control distribution (19 measurements). Blue: crewmember A; red crewmember B. **P ≤ 0.01 (*z*-score test).

**Figure 3:**
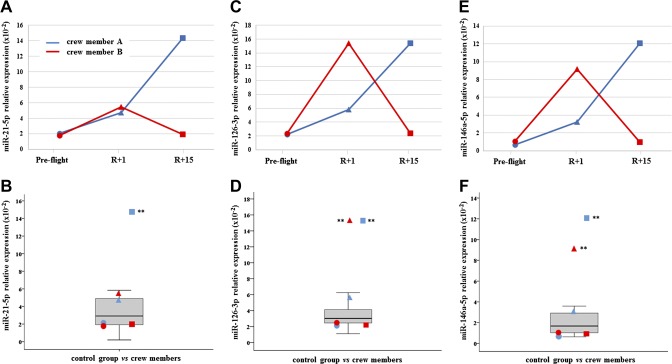
C-inflamma-miRs-21-5p; -126-3p and -146a-5p. *A*, *C*, *E*) Measurements of circulating inflamma-miRs-21-5p (*A*); -126-3p (*C*), and -146a-5p (*E*) are reported in dependence of time in both crewmembers. For explanation of symbols see Fig. 1. *B*, *D*, *F*) C-inflamma-miRs-21-5p (*B*); -126-3p (*D*), and -146a-5p (*F*) values of crewmembers are compared with age-matched control distribution (19 measurements). Blue: crewmember A; red crewmember B. **P ≤ 0.01 (*z*-score test).

**Figure 4:**
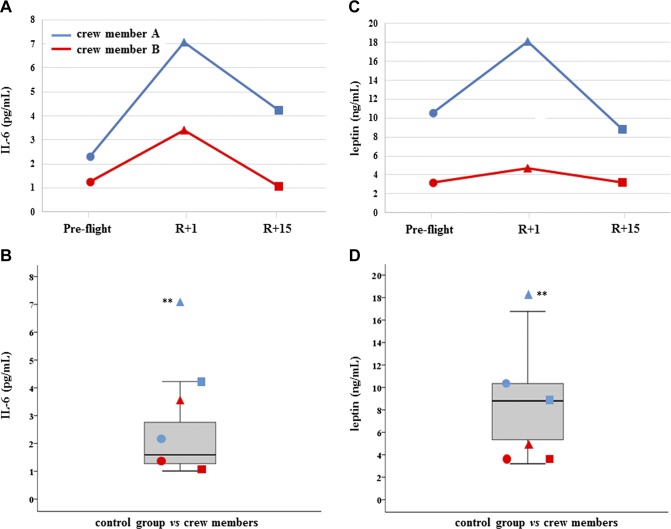
IL-6 and leptin. *A*, *C*) Measurements of circulating IL-6 (*A*) and leptin (*C*) are, reported in dependence of time in both crewmembers. For explanation of symbols see Fig. 1. *B*, *D*) IL-6 (*B*) and leptin (*D*) values of crewmembers are compared with age-matched control distribution (19 measurements). Blue: crewmember A; red crewmember B. **P ≤ 0.01 (*z*-score test).

All results have been reported both in dependence of time series (preflight, R+1, and R+15) and in comparison with the control distribution. C-proteasome was found significantly increased in both crewmembers at R+1, but subject A had not recovered at R+15 and c-proteasome resulted in further increase, whereas subject B did recover (**Fig. 1**). Myo-miR-206 was found significantly increased in crewmember A at both R+1 and R+15, whereas was increased only at final recovery time in crewmember B (**Fig. 2**). On the contrary, myo-miR-133a-3p showed no significant changes in both crewmembers when compared with control distribution (Supplemental Fig. S1*A*, *B*). Inflamma-miR-21-5p was significantly increased in crewmember A at R+15, whereas the changes revealed in crewmember B were not significant (**Fig. 3*A, B***). Similarly, inflamma-miR-126-3p was significantly increased in crewmember A at R+1, whereas the significant increase in crewmember B (at R+1) was completely recovered (Fig. 3*C, D*). Similar results were obtained when measuring inflamma-miR-146a-5p in both crewmembers (Fig. 3*E, F*). The c-level of miR-122-5p was found significantly increased at R+15 in crewmember A only (Supplemental Fig. S1*C*, *D*). MiR-145-5p was observed to be increased at landing time in both crewmembers, but only subject B completely recovered (Supplemental Fig. S2*A*, *B*). The different trends of miR-363-3p observed in both crewmembers were not significant when compared with control group (Supplemental Fig. S2*C*, *D*). The changes of TGF-β1 observed in the 2 crewmembers were not significant (Supplemental Fig. S3*A*, *B*), and similarly, the c-mtDNA was not found significantly modified in the 2 crewmembers (Supplemental Fig. S3*C*, *D*), even if values were at the extreme of control distribution. Dissimilarly, IL-6 and leptin were significantly increased at R+1 and recovered in crewmember A, whereas they did not change in crewmember B (**Fig. 4*A, D***).

**Figure 5:**
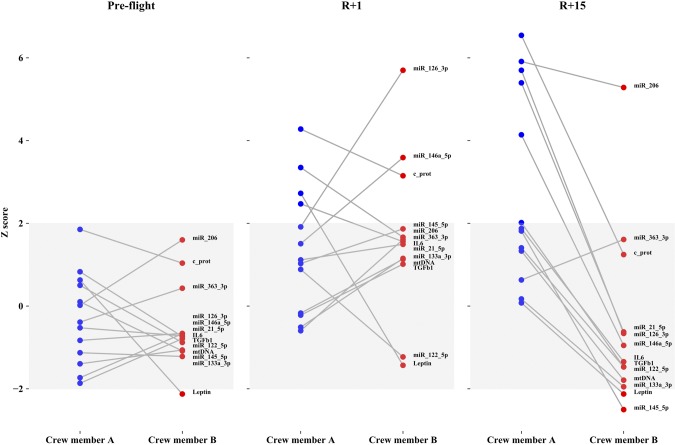
All normalized markers in crewmembers A and B at preflight, R+1, and R+15 times. *Y*-axis describes *z* scores and gray zone contains control group values. Values outside the gray zone are considered significant.

**Figure 6:**
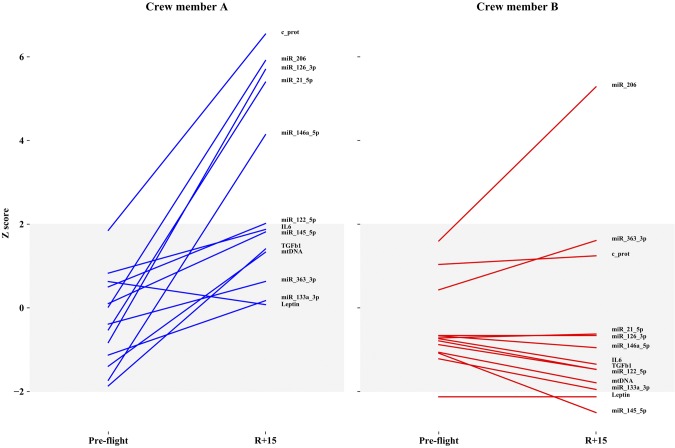
The 2 crewmembers, A and B, are compared at preflight *vs.* R+15. All markers are normalized. *Y*-axis describes *z* scores and gray zone contains control group values. Values outside the gray zone are considered significant.

The exploitation of normalized data is reported in **Figs. 5** and **6** by graphing the *z* score of each molecular target compared with the baseline population (gray bands indicate 95% of the population distribution). Figure 5 shows the differences between the 2 astronauts at the 3 times, whereas Fig. 6 shows the difference between the 2 astronauts considering only 2 times (*i.e.*, preflight and R+15). The normalized values of circulating molecules resulted in the normal distribution at the baseline, and differences were observed after landing and recovery for each crewmember. Many differences were observed between the 2 subjects at R+1 in terms of inflamma-miRs, myo-miR-206, c-proteasome, and IL-6/leptin, thus making the 2 crewmembers dissimilar to each other. Examining the *z* scores on the same astronaut before flight and after recovery (Fig. 6), the baseline values of most parameters are already inside the limits of the standard distribution of the population. The only molecule that significantly varies in both astronauts after spaceflight is myo-miR-206. Dissimilarly, c-proteasome, inflamma-miRs, and myo-miR-206 were not reverted to the baseline values at R+15 in crewmember A.

A bioinformatic approach was applied to investigate currently validated and miR-target union of all inflamma-miRs-21-5p, -126-3p, -146a-5p and separately, myo-miR-206. Two lists of 331 and 136 genes/transcripts were reported in 2 supplemental tables, respectively (Supplemental Tables S1 and S2). In particular, the former table is referred to GO: response to stress (*P* = 6.78206035218e−33), whereas the latter is specifically related to miR-206 transcript targets.

**Figure 7:**
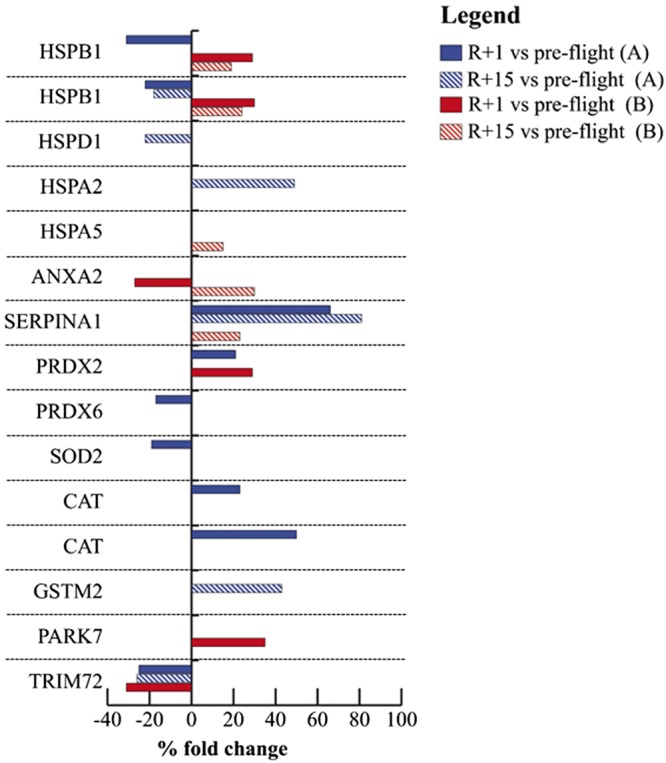
Proteomic analysis in human skeletal muscle. Histograms of stress and antioxidant proteins differentially expressed in the soleus muscle between baseline *vs.* R+1 (colored bars) and baseline *vs.* R+15 (striped bars) in crewmember A (blue bars) and B (red bars), as detected by 2-D-DIGE analysis. Proteins significantly changed (paired 1-way ANOVA and Tukey’s test, α = 0.01) are indicated by their gene name and expressed as a percent of spot volume variation. Statistical details are showed in Table 2.

Muscle proteomic analyses, tested for evaluable miR-targets, indicated significant differences in stress and antioxidant proteins comparing baseline with postflight (R+1) in 9 and 6 spots in crewmember A and B, respectively. Then, comparing baseline to recovery time (R+15), 6 spots changed in crewmember A and 5 spots in crewmember B (**Fig. 7** and Table 2). In particular, 2 proteoforms (21), different molecular forms originated from the heat shock protein family B (small) member 1 gene (*HSPB1*) were changed in abundance in both crewmembers at R+1 (−31 and −22% in A; +29 and +30% in B) and recovery (−18% in A; +19 and +24% in B). Tripartite motif-containing protein 72 (TRIM72) was down-regulated after landing in both crewmembers (−25% in A and −31% in B) and only in A at recovery (−26%). SERPINA1 was more abundant in A after landing (+66%) and in both at recovery (+81% in A and +23% in B). SERPINA1 is also a validated target of miR-126-3p (22). Annexin A2 (ANXA2, validated target of miR-146a-5p) was down-regulated at R+1 (−27%) and up-regulated at recovery (+30%) in crewmember B. Peroxiredoxin-2 (PRDX2) was more abundant in both crewmembers postflight (R+1) (+21% in A; +29% in B). Peroxiredoxin-6 (PRDX6) and superoxide dismutase 2 (SOD2, a validated target of miR-21-5p and -146a-5p) were decreased (−17 and −19%), whereas 2 proteoforms of catalase were increased (+23 and +50%) in crewmember A after landing. Heat shock–related 70 kDa protein 2 (HSPA2, +49%) and glutathione S-transferase Mu 2 (GSTM2, +43%, putative target of miR-21-5p) were increased in abundance, whereas the mitochondrial 60 kDa heat shock protein (HSPD1, −22%) was down-regulated in crewmember A at recovery time. Protein/nucleic acid deglycase DJ-1 (PARK7, +35%, validated target of miR-126-3p) and endoplasmic reticulum chaperone BiP (HSPA5, +15%, validated target of miR-21-5p) were increased in crewmember B at R+1 and recovery, respectively.

## DISCUSSION

Prolonged distress or chronic exposures to stressors, including psychologic or physical stresses, are known to affect immune system function, which in turn increases inflammatory mediators (23, 24). Furthermore, chronic stress throughout the lifespan in absence or modest bodily adaptation and inefficient repair mechanisms may affect the ageing process and lifespan (15, 25, 26), favoring the disease onset (27, 28). Spaceflight may also represent a source of prolonged/chronic stress that is due not only to the psychologic aspect but also environment stressors, such as adaptation to microgravity, high workload, sleep deprivation, isolation and confinement, ionizing radiation, and potentially others (29).

The present work aimed at answering the questions of whether the 2 crewmembers (A and B), beyond the effects on skeletal muscle (9), had systemic effects in terms of pro- and anti-inflammatory c-molecules after about 6 mo of spaceflight at the ISS and if they recovered at 1 or 15 d after landing.

Both atrophy of skeletal muscle and systemic stress may affect the entire body, being that skeletal muscle is the most abundant tissue of the human body (about 30–40% of the body) and systemic stress is able to alter metabolism and homeostasis (3, 30, 31). Furthermore, recent evidence supports the hypothesis that systemic stress-evoked sterile inflammation initiates by the sympathetic nervous system, resulting in the increase of c-damage associated molecular patterns, such as mtDNA, and a reduction in immune-inhibitory miRs, which are carried in the blood circulation to tissues throughout the body (32).

To examine at the systemic level possible unbalancing homeostasis in terms of pro- and anti-inflammatory molecules, we measured relevant blood molecules and epigenetic regulators (*i.e.*, c-myo-miRs-206 and -133a-3p, c-inflamma-miRs -21-5p, -126-3p, and -146a-5p, liver c-miR-122-5p, cell proliferation regulators c-miR-145-5p and -363-3p, c-proteasome, c-mtDNA, proinflammatory IL-6, anti-inflammatory TGF-β1, and leptin). Indeed, types of various c-shuttles (nano-microextracellular vesicles, proteins, and apolipoproteins) were not the objective of the present work, whereas the measurement of the total amount of c-miRs/different molecules was supposed to be more significant.

Relevant differences between the 2 crewmembers at 1 or 15 d, or both points, of recovery were identified, even if these findings cannot define the in-flight c-levels of the same molecules. Crewmember A showed more deviations from baseline after landing time (R+1) than crewmember B. In fact, c-inflamma-miRs -21-5p, -126-3p, and -146a-5p, and myo-miR-206, c-proteasome, IL-6, and leptin were significantly increased after 1 d recovery. At R+15, crewmember A showed a significant increase of c-proteasome, inflamma-miRs, and myo-miR-206. Comparing these data with crewmember B, the only common molecule was myo-miR-206, which was still increased at R+15 in both crewmembers. It is known that myo-miR-206 is preferentially expressed in skeletal muscle and completely absent, or expressed at relatively low levels, in other tissues. Notably, crewmember B trained more vigorously than A, particularly concerning the loading forces. In the postflight, crewmember A showed substantial decrements (*i.e.*, muscle volume and architecture) in strength and in fiber contractility, which was strongly mitigated in B, as previously reported in a separate work on the same individuals (9). In fact, the increased level of c-miR-206 at landing time in astronaut A and in both astronauts at R+15 may also be associated with the different physical training status of the subjects. This finding suggests a possible role of c-miR-206 as a good candidate for the monitoring of skeletal muscle status. Regardless, a consistent literature indicates the full involvement of myo-miR-206 in different conditions, such as age, physical training, and type of exercise, such as acute or prolonged, aerobic or resistance or endurance activity (33–36).

MiR-206 promotes cell differentiation and cell inhibition and may influence cell regeneration in the muscle (37). In particular, miR-206 and miR-21 have been found to increase in muscle tissue in catabolic/atrophy condition in a mouse model (38). The contribution of muscle atrophy/wasting to the pool of c-miRs was recently confirmed in exosomes released by myofibers, supporting the conclusion that myofiber-derived exosomes modulate protein levels of key factors in myogenic or osteogenic differentiation of mesenchymal progenitor cells (39). In particular, miR-21-5p was shown to promote the osteogenic differentiation of mouse bone marrow cells by targeting Sprouty homolog 1 (Spry1), negatively regulating the osteogenic differentiation of mesenchymal stem cells (40).

As far as inflamma-miRs are concerned, crewmember A showed the highest levels at R+15, whereas crewmember B showed increases of miR-126-3p and -146a-5p only at R+1, thus revealing 2 different trends between the 2 subjects, those being regulators of both stress response and inflammatory pathway (8).

Cellular miRs were previously studied in both *in vitro* microgravity experiments on earth and *in vitro* experiments run in the ISS. The former study was conducted with γ-ray coexposure and many miRs involving cell cycle machinery and DNA repair system resulted dysregulated (41). The latter showed the dysregulation of miR-21 in a different experiment setting (42), thus highlighting its involvement in spaceflight effects.

Once c-inflamma-miRs are up-taken by cells and tissue, they are able to modulate many genes. Taking into account KEGG pathway analysis, NF-κB pathway had high significance (*P* = 2.516315e−08), but p53 signaling (*P* = 9.370703e−07) and the mechanistic target of rapamycin (mTOR) pathway (*P* = 0.0001298907) also significantly fit the miR-targets. In particular, phosphatase and tensin homolog (PTEN), phosphatidylinositol 3-kinase regulatory subunit α (PIK3R1), phosphatidylinositol 3-kinase regulatory subunit β (PIK3R2), insulin receptor substrate 1 (IRS1), and serine/threonine kinase 1 (AKT1) are inflamma-miR targets and represent the central pathway involving muscle/tissue anabolism/synthesis. Accordingly, some proteins related to mTOR pathway have also been identified as dysregulated in our previous paper (9). Taking into account the GO category analysis, stress response resulted among those strongly significant (*P* = 6.78206035218e−33). Interestingly, a common target of both miR-21-5p and miR-146a-5p is CLOCK (43, 44). This protein plays a central role in the regulation of circadian rhythms and could have a systemic role because of its ubiquitous expression in testis, thyroid, and many other tissues.

The inflamma-miR increase may be due to an augmented exocytosis that can be, at least in part, stress related (32), due to an increased tissue and cell injury, or both, especially in crewmember A. The tissue injury is also confirmed by the increase of c-proteasome, reaching a concentration similar to that of autoimmune disease (45) in crewmember A after final recovery. Accordingly, the increase of c-mtDNA was dramatically evident in crewmember A, even if at the limit of the normal range. Overall, the *in vivo* c-proteasome and c-mtDNA levels mediate the inflammatory pathway and represent a general mechanism to switch on inflammation, immune cell activities also being markers of muscle wasting (16, 46). On the other side, the increase of c-proteasome in crewmember A at R+15 d as a marker of muscle recovery cannot be completed excluded (47). Noteworthy, IL-6 increased only in astronaut A at 1 d recovery concomitantly to leptin, and both are important regulators of inflammation and bone turnover (48).

Importantly, various stress-related and antioxidant proteins were found modified in skeleton muscle, and many of them are direct targets of c-inflamma-miRs. A direct or indirect effect between muscle tissue proteins and blood c-miRs may only be speculated, which is a limitation of the work. However, it is worth noting that concomitantly with the increased inflamma-miR levels in astronaut A, the soleus muscle tissue showed an increase of 80% α SERPINA1, a serine protease inhibitor belonging to acute phase protein and validated target of miR-126-3p (22), apparently as a tissue-related anti-inflammatory response. This effect was also revealed in astronaut B but to a lesser extent and at final recovery time only. Recent data suggest that SERPINA1 is also expressed by endothelial cells after exposure to simulated microgravity (49) and may represent an important marker of tissue-related anti-inflammatory response. SERPINA1 increase can be due to the miR-126-3p decrease, especially in endothelial cells where it is usually expressed (50), assuming that a relationship exists with the miR-126-3p increase in the blood, as observed in both astronauts even if timing differed.

In agreement with all data obtained, spaceflight recovery had greater effects on crewmember A than B. In fact, muscle stress–related proteins, such as HSPs, GSTM, PRDX, ANXA2, and PARK7, were largely modified in crewmember A rather than in B. These results point to muscle-stress responses that also involve oxido-reductase enzymes like SOD and catalase as well as the repair membrane protein TRIM72. The latter was decreased in muscle tissue at both recovery times in astronaut A and at final recovery time in astronaut B. Similar results in terms of stress-related pathway activation were previously observed in mouse model after 91 d of spaceflight (51).

Overall, these results corroborate the view obtained from our previous paper (9), where the 2 crewmembers had different spaceflight recovery effects, in which crewmember A was the most affected. In particular, muscle-related stress and proinflammatory status are here highlighted in crewmember A, whereas crewmember B’s recovery was almost completed except for myo-miR-206. Inflamma-miRs seem to mediate the systemic recovery of crewmember A, and they are expected to reach a complete recovery beyond 15 d from landing.

In a complex field like space and spaceflight, N of 1 could be a critical issue. However, population size, usually based on a relatively low number of crewmembers, can be overwhelmed by time series (or longitudinal) personalized studies. In fact, the effects of the 6-mo chronic exposure to such an environment with many variables can have substantially different effects in different individuals, thus the study of intraindividual variability along the time of exposure/recovery becomes more informative (52).

Based on a personalized time series analysis, the present data further underpin the importance of countermeasures aimed at reducing, as much as possible, skeletal muscle wasting (9). Moreover, these data further suggest a linkage among muscle wasting, stress response and inflammation and potentially affecting systemic metabolism. In this perspective, the prolonged or chronic exposure to space/spaceflight may favor the development of metabolic alterations, even if additional analyses with later time points are necessary.

## Supplementary Material

This article includes supplemental data. Please visit *http://www.fasebj.org* to obtain this information.

## Abbreviations

2-D: 2-dimensional
GO: Gene Ontology
ISS: International Space Station
miR: microRNA
MS/MS: tandem mass spectrometry
mtDNA: mitochondrial DNA
myo: muscle specific
OD: optical density
PMF: peptide mass fingerprinting
preflight: 76–79 d before flight
SERPINA1: α-1-antitrypsin
