# Supplementary figures and images for: Recovery from 6-month spaceflight at the International Space Station: muscle-related stress into a proinflammatory setting
Source: FASEB J. 2019 Jan 8;33(4):5168–80. doi: 10.1096/fj.201801625R (PMC6436655; doi:10.1096/fj.201801625R)

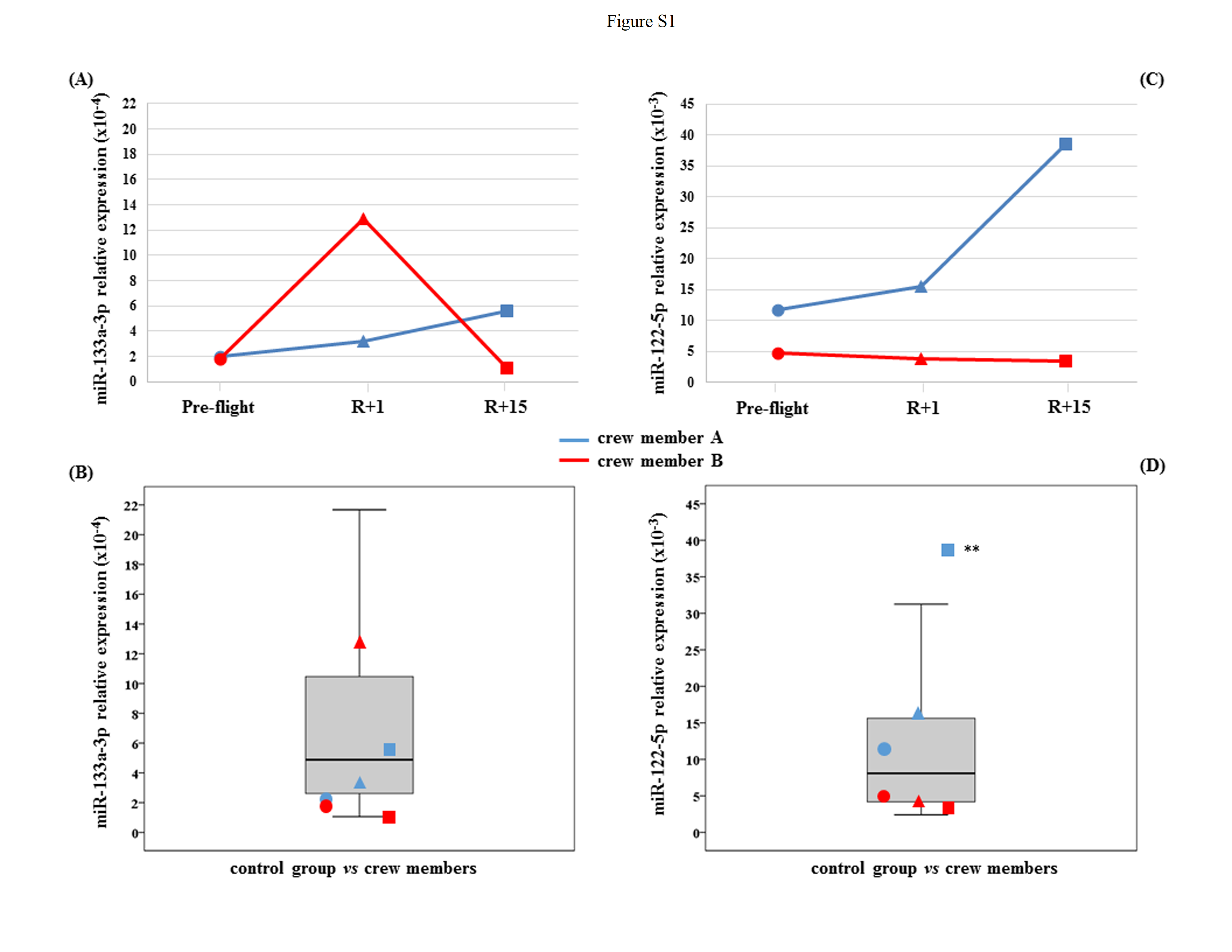

Supplement: Supplementary file 1 [file fj.201801625R.sf1.tif]

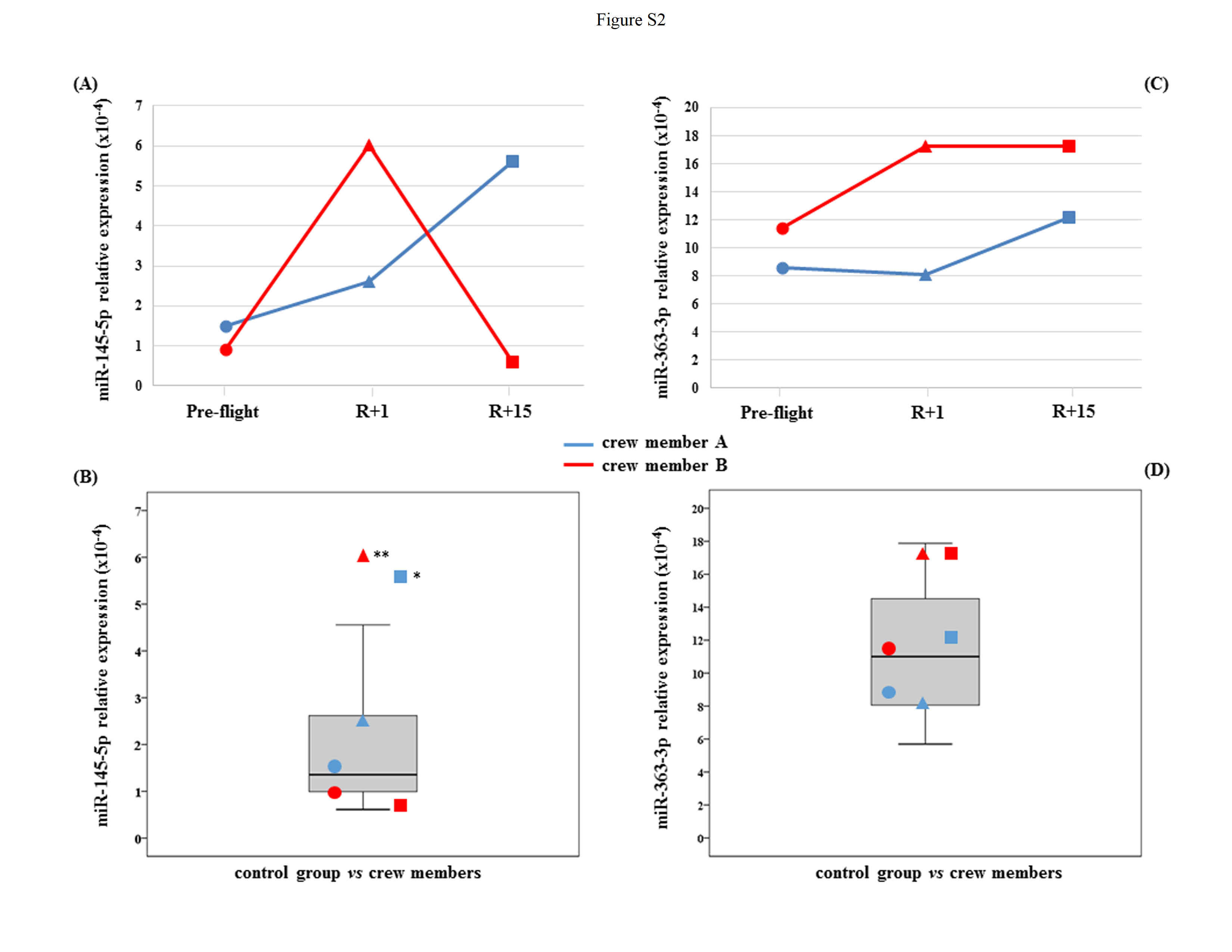

Supplement: Supplementary file 2 [file fj.201801625R.sf2.tif]

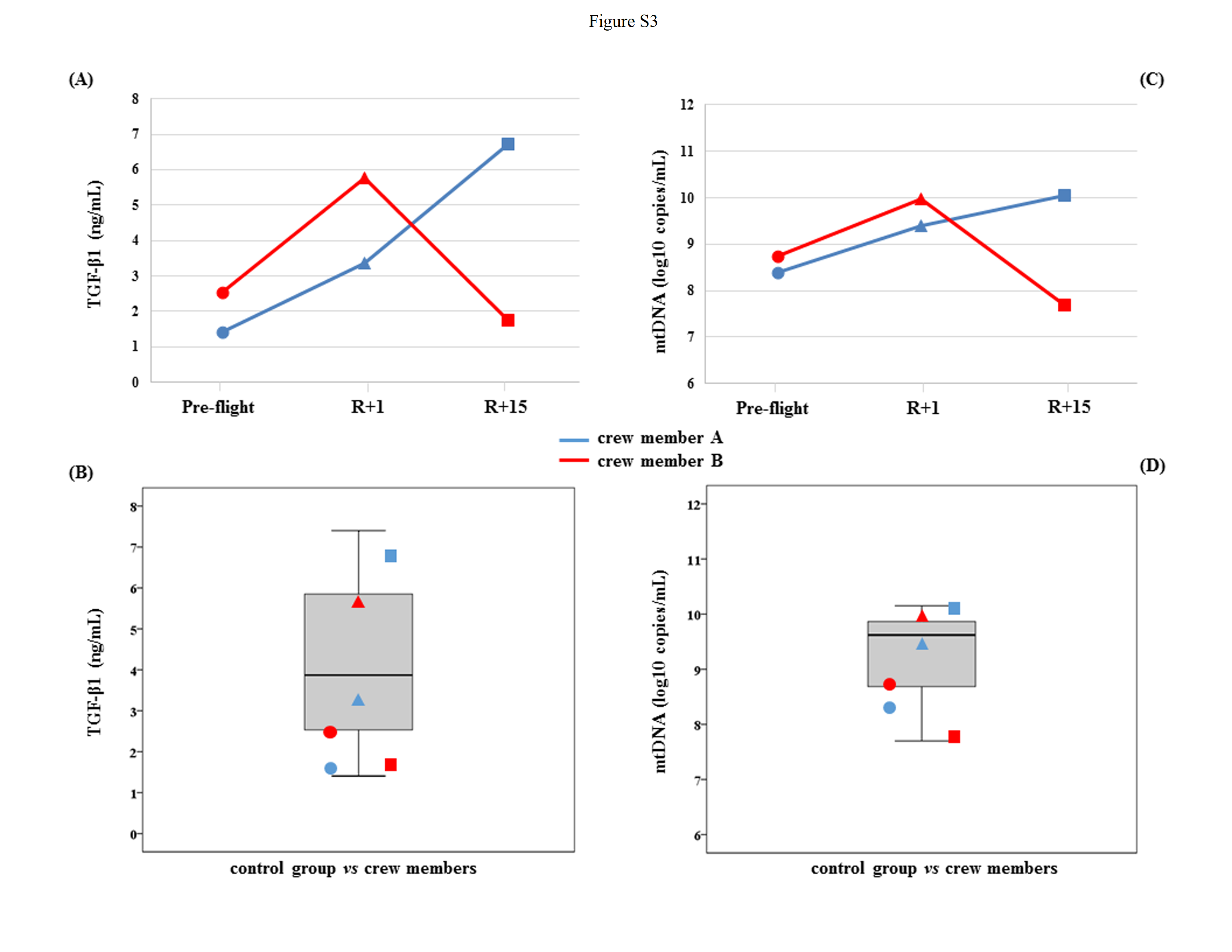

Supplement: Supplementary file 3 [file fj.201801625R.sf3.tif]
