# Supplementary material for: Recovery from 6-month spaceflight at the International Space Station: muscle-related stress into a proinflammatory setting
Source: FASEB J. 2019 Jan 8;33(4):5168–80. doi: 10.1096/fj.201801625R (PMC6436655; doi:10.1096/fj.201801625R)
Supplement: Supplementary file 4 [file fj.201801625R.sf4.pptx]

## Slide 1
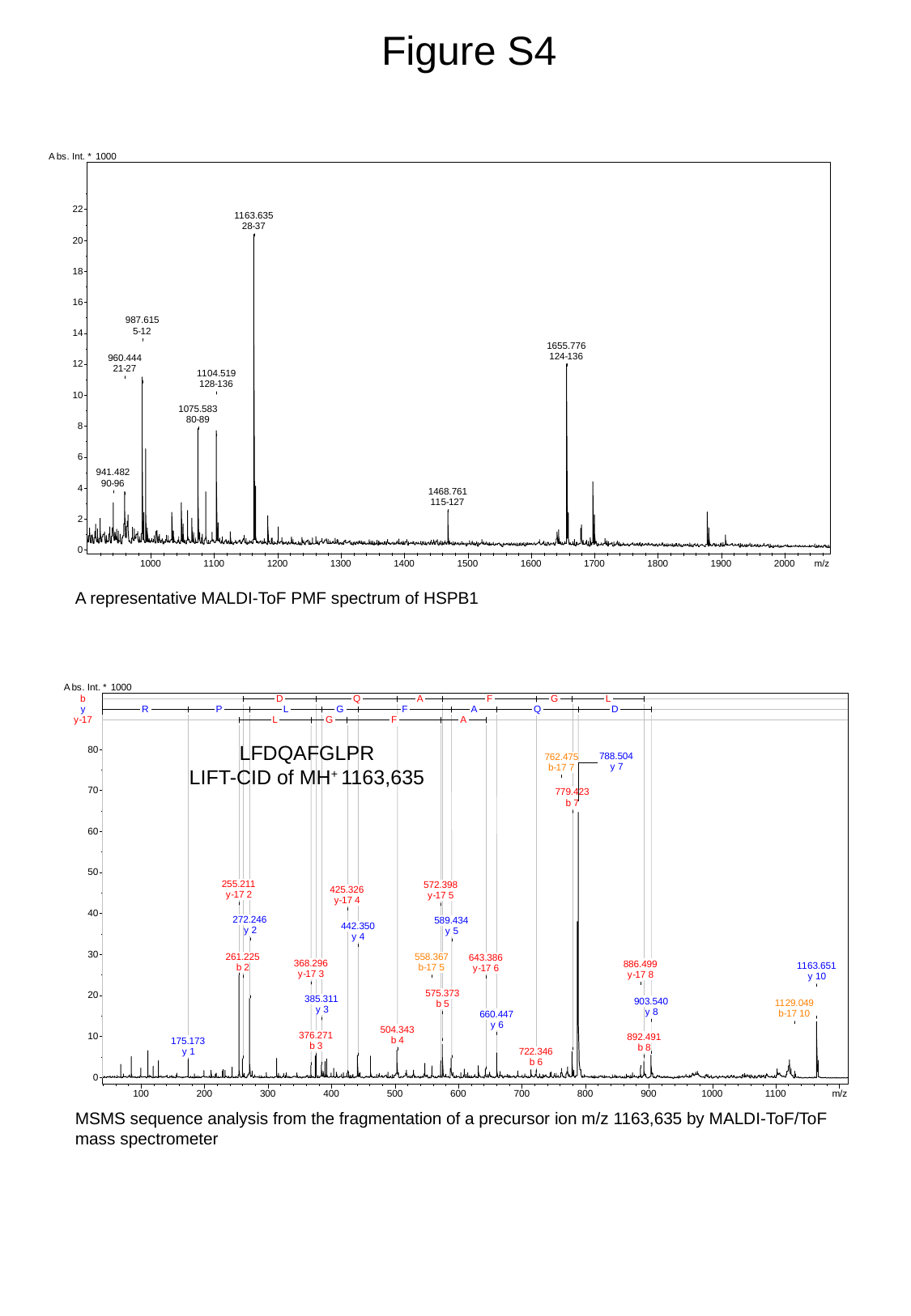

# Figure S4
A representative MALDI-ToF PMF spectrum of HSPB1
LFDQAFGLPR
LIFT-CID of MH+ 1163,635
MSMS sequence analysis from the fragmentation of a precursor ion m/z 1163,635 by MALDI-ToF/ToF mass spectrometer
