# Supplementary material for: Recovery from 6-month spaceflight at the International Space Station: muscle-related stress into a proinflammatory setting
Source: FASEB J. 2019 Jan 8;33(4):5168–80. doi: 10.1096/fj.201801625R (PMC6436655; doi:10.1096/fj.201801625R)
Supplement: Supplementary file 5 [file fj.201801625R.st1.docx]

**Table S1.** List of validated genes (transcripts) targeted by inflamma-miRs. GO analysis: response to stress (p = 6.78206035218e-33).

| **#** | **Gene Name** | **Gene Ensembl id** |
| --- | --- | --- |
| **1.** | **PELI1** | ENSG00000197329 |
| **2.** | **ATF6** | ENSG00000118217 |
| **3.** | **BRK1** | ENSG00000254999 |
| **4.** | **IRS2** | ENSG00000185950 |
| **5.** | **ADAM9** | ENSG00000168615 |
| **6.** | **ACTB** | ENSG00000075624 |
| **7.** | **TLR2** | ENSG00000137462 |
| **8.** | **ZMAT3** | ENSG00000172667 |
| **9.** | **HIPK2** | ENSG00000064393 |
| **10.** | **GSK3B** | ENSG00000082701 |
| **11.** | **SOX4** | ENSG00000124766 |
| **12.** | **MARC1** | ENSG00000186205 |
| **13.** | **RPS3** | ENSG00000149273 |
| **14.** | **CCAR2** | ENSG00000158941 |
| **15.** | **E2F1** | ENSG00000101412 |
| **16.** | **HSPA1A** | ENSG00000204389 |
| **17.** | **TGFBR1** | ENSG00000106799 |
| **18.** | **ERBB2** | ENSG00000141736 |
| **19.** | **APEX1** | ENSG00000100823 |
| **20.** | **NFKB1** | ENSG00000109320 |
| **21.** | **TIPIN** | ENSG00000075131 |
| **22.** | **IRF4** | ENSG00000137265 |
| **23.** | **SMC1A** | ENSG00000072501 |
| **24.** | **UBXN4** | ENSG00000144224 |
| **25.** | **POLR1C** | ENSG00000171453 |
| **26.** | **YAP1** | ENSG00000137693 |
| **27.** | **WASL** | ENSG00000106299 |
| **28.** | **KAT6A** | ENSG00000083168 |
| **29.** | **GCLC** | ENSG00000001084 |
| **30.** | **LTA4H** | ENSG00000111144 |
| **31.** | **MAP4K2** | ENSG00000168067 |
| **32.** | **UBE2T** | ENSG00000077152 |
| **33.** | **CXCL8** | ENSG00000169429 |
| **34.** | **ATF2** | ENSG00000115966 |
| **35.** | **TPM1** | ENSG00000140416 |
| **36.** | **EIF2AK3** | ENSG00000172071 |
| **37.** | **RNASEL** | ENSG00000135828 |
| **38.** | **CXCR4** | ENSG00000121966 |
| **39.** | **AMFR** | ENSG00000159461 |
| **40.** | **NRAS** | ENSG00000213281 |
| **41.** | **PRKAA2** | ENSG00000162409 |
| **42.** | **CRKL** | ENSG00000099942 |
| **43.** | **CD80** | ENSG00000121594 |
| **44.** | **BID** | ENSG00000015475 |
| **45.** | **SRPRB** | ENSG00000144867 |
| **46.** | **CCNA2** | ENSG00000145386 |
| **47.** | **BAIAP2** | ENSG00000175866 |
| **48.** | **YOD1** | ENSG00000180667 |
| **49.** | **APC** | ENSG00000134982 |
| **50.** | **CRK** | ENSG00000167193 |
| **51.** | **GATA3** | ENSG00000107485 |
| **52.** | **CFL1** | ENSG00000172757 |
| **53.** | **UBR5** | ENSG00000104517 |
| **54.** | **TRIAP1** | ENSG00000170855 |
| **#** | **Gene Name** | **Gene Ensembl id** |
| **55.** | **THBS1** | ENSG00000137801 |
| **56.** | **HSPA4** | ENSG00000170606 |
| **57.** | **IL1B** | ENSG00000125538 |
| **58.** | **IL12A** | ENSG00000168811 |
| **59.** | **CUL2** | ENSG00000108094 |
| **60.** | **PAK2** | ENSG00000180370 |
| **61.** | **ATMIN** | ENSG00000166454 |
| **62.** | **CCR9** | ENSG00000173585 |
| **63.** | **C11orf30** | ENSG00000158636 |
| **64.** | **EDEM2** | ENSG00000088298 |
| **65.** | **MAP2K7** | ENSG00000076984 |
| **66.** | **IFITM1** | ENSG00000185885 |
| **67.** | **IFIT1** | ENSG00000185745 |
| **68.** | **WFS1** | ENSG00000109501 |
| **69.** | **USP1** | ENSG00000162607 |
| **70.** | **WNT5A** | ENSG00000114251 |
| **71.** | **METRNL** | ENSG00000176845 |
| **72.** | **XRCC6** | ENSG00000196419 |
| **73.** | **PIK3R2** | ENSG00000105647 |
| **74.** | **MCL1** | ENSG00000143384 |
| **75.** | **CD83** | ENSG00000112149 |
| **76.** | **PSMD2** | ENSG00000175166 |
| **77.** | **ANXA1** | ENSG00000135046 |
| **78.** | **FOXM1** | ENSG00000111206 |
| **79.** | **HERPUD1** | ENSG00000051108 |
| **80.** | **ZNF148** | ENSG00000163848 |
| **81.** | **BACH1** | ENSG00000156273 |
| **82.** | **HUWE1** | ENSG00000086758 |
| **83.** | **FRS2** | ENSG00000166225 |
| **84.** | **ITCH** | ENSG00000078747 |
| **85.** | **STK11** | ENSG00000118046 |
| **86.** | **REV1** | ENSG00000135945 |
| **87.** | **NBR1** | ENSG00000188554 |
| **88.** | **CD40LG** | ENSG00000102245 |
| **89.** | **HSPA5** | ENSG00000044574 |
| **90.** | **SOD1** | ENSG00000142168 |
| **91.** | **ISG15** | ENSG00000187608 |
| **92.** | **SMC5** | ENSG00000198887 |
| **93.** | **BCL2** | ENSG00000171791 |
| **94.** | **MAP2K3** | ENSG00000034152 |
| **95.** | **TUBB** | ENSG00000196230 |
| **96.** | **MACF1** | ENSG00000127603 |
| **97.** | **TP63** | ENSG00000073282 |
| **98.** | **CCR6** | ENSG00000112486 |
| **99.** | **AGO2** | ENSG00000123908 |
| **100.** | **TMX1** | ENSG00000139921 |
| **101.** | **MBTPS2** | ENSG00000012174 |
| **102.** | **BRCA2** | ENSG00000139618 |
| **103.** | **PPP2R5D** | ENSG00000112640 |
| **104.** | **TNFAIP3** | ENSG00000118503 |
| **105.** | **PSMD3** | ENSG00000108344 |
| **106.** | **RPS27A** | ENSG00000143947 |
| **107.** | **EGFR** | ENSG00000146648 |
| **108.** | **SRPK2** | ENSG00000135250 |
| **#** | **Gene Name** | **Gene Ensembl id** |
| **109.** | **AGO4** | ENSG00000134698 |
| **110.** | **LY75** | ENSG00000054219 |
| **111.** | **IFITM3** | ENSG00000142089 |
| **112.** | **SQSTM1** | ENSG00000161011 |
| **113.** | **USP47** | ENSG00000170242 |
| **114.** | **SNN** | ENSG00000184602 |
| **115.** | **MAP3K1** | ENSG00000095015 |
| **116.** | **TLR4** | ENSG00000136869 |
| **117.** | **HAS2** | ENSG00000170961 |
| **118.** | **RAB1A** | ENSG00000138069 |
| **119.** | **PDCD6** | ENSG00000249915 |
| **120.** | **TAB2** | ENSG00000055208 |
| **121.** | **HSPH1** | ENSG00000120694 |
| **122.** | **KRAS** | ENSG00000133703 |
| **123.** | **DDX3X** | ENSG00000215301 |
| **124.** | **PARP9** | ENSG00000138496 |
| **125.** | **TGFB1** | ENSG00000105329 |
| **126.** | **PRKCE** | ENSG00000171132 |
| **127.** | **TAOK1** | ENSG00000160551 |
| **128.** | **TAB3** | ENSG00000157625 |
| **129.** | **CAPZA1** | ENSG00000116489 |
| **130.** | **DDX60** | ENSG00000137628 |
| **131.** | **SOCS6** | ENSG00000170677 |
| **132.** | **TNRC6B** | ENSG00000100354 |
| **133.** | **FADD** | ENSG00000168040 |
| **134.** | **SREBF2** | ENSG00000198911 |
| **135.** | **NMI** | ENSG00000123609 |
| **136.** | **RFC1** | ENSG00000035928 |
| **137.** | **TRIP12** | ENSG00000153827 |
| **138.** | **HSPA1A** | ENSG00000215328 |
| **139.** | **SLFN11** | ENSG00000172716 |
| **140.** | **PTK2** | ENSG00000169398 |
| **141.** | **DUSP10** | ENSG00000143507 |
| **142.** | **MAVS** | ENSG00000088888 |
| **143.** | **BRCA1** | ENSG00000012048 |
| **144.** | **TRIM22** | ENSG00000132274 |
| **145.** | **TAF1** | ENSG00000147133 |
| **146.** | **TMX4** | ENSG00000125827 |
| **147.** | **AGO1** | ENSG00000092847 |
| **148.** | **CHORDC1** | ENSG00000110172 |
| **149.** | **POLR2A** | ENSG00000181222 |
| **150.** | **BTG2** | ENSG00000159388 |
| **151.** | **C1RL** | ENSG00000139178 |
| **152.** | **PIK3CD** | ENSG00000171608 |
| **153.** | **JUN** | ENSG00000177606 |
| **154.** | **BABAM1** | ENSG00000105393 |
| **155.** | **TOP2A** | ENSG00000131747 |
| **156.** | **CCND1** | ENSG00000110092 |
| **157.** | **SMAD4** | ENSG00000141646 |
| **158.** | **CTNNB1** | ENSG00000168036 |
| **159.** | **MSH6** | ENSG00000116062 |
| **160.** | **ASNS** | ENSG00000070669 |
| **161.** | **RASGRP1** | ENSG00000172575 |
| **162.** | **OASL** | ENSG00000135114 |
| **163.** | **DOCK1** | ENSG00000150760 |
| **164.** | **IFIT3** | ENSG00000119917 |
| **165.** | **AXL** | ENSG00000167601 |
| **#** | **Gene Name** | **Gene Ensembl id** |
| **166.** | **TRAF6** | ENSG00000175104 |
| **167.** | **AKT1** | ENSG00000142208 |
| **168.** | **TLK2** | ENSG00000146872 |
| **169.** | **CDK9** | ENSG00000136807 |
| **170.** | **CREBRF** | ENSG00000164463 |
| **171.** | **F2R** | ENSG00000181104 |
| **172.** | **ACTR2** | ENSG00000138071 |
| **173.** | **SUPT16H** | ENSG00000092201 |
| **174.** | **FOXN3** | ENSG00000053254 |
| **175.** | **MYC** | ENSG00000136997 |
| **176.** | **SRPK1** | ENSG00000096063 |
| **177.** | **IFI44L** | ENSG00000137959 |
| **178.** | **RAD23A** | ENSG00000179262 |
| **179.** | **TP53INP1** | ENSG00000164938 |
| **180.** | **UBA1** | ENSG00000130985 |
| **181.** | **MSH2** | ENSG00000095002 |
| **182.** | **CCL20** | ENSG00000115009 |
| **183.** | **ATRX** | ENSG00000085224 |
| **184.** | **POLR2E** | ENSG00000099817 |
| **185.** | **ECT2** | ENSG00000114346 |
| **186.** | **CD86** | ENSG00000114013 |
| **187.** | **WASF2** | ENSG00000158195 |
| **188.** | **HSP90AB1** | ENSG00000096384 |
| **189.** | **MIA3** | ENSG00000154305 |
| **190.** | **ZNF175** | ENSG00000105497 |
| **191.** | **HSF2** | ENSG00000025156 |
| **192.** | **PTGS2** | ENSG00000073756 |
| **193.** | **MUC1** | ENSG00000185499 |
| **194.** | **MR1** | ENSG00000153029 |
| **195.** | **PIK3R1** | ENSG00000145675 |
| **196.** | **AEN** | ENSG00000181026 |
| **197.** | **IFIT5** | ENSG00000152778 |
| **198.** | **CD97** | ENSG00000123146 |
| **199.** | **RORA** | ENSG00000069667 |
| **200.** | **HSP90B1** | ENSG00000166598 |
| **201.** | **TBK1** | ENSG00000183735 |
| **202.** | **EPHA2** | ENSG00000142627 |
| **203.** | **IRS1** | ENSG00000169047 |
| **204.** | **PRKAA1** | ENSG00000132356 |
| **205.** | **MAP7** | ENSG00000135525 |
| **206.** | **PEA15** | ENSG00000162734 |
| **207.** | **SESN1** | ENSG00000080546 |
| **208.** | **HSPA4L** | ENSG00000164070 |
| **209.** | **TNFRSF10B** | ENSG00000120889 |
| **210.** | **PSMC3** | ENSG00000165916 |
| **211.** | **HSPA8** | ENSG00000109971 |
| **212.** | **NF1** | ENSG00000196712 |
| **213.** | **YY1** | ENSG00000100811 |
| **214.** | **MDM4** | ENSG00000198625 |
| **215.** | **BCL6** | ENSG00000113916 |
| **216.** | **FANCF** | ENSG00000183161 |
| **217.** | **FAS** | ENSG00000026103 |
| **218.** | **RPS6KA3** | ENSG00000177189 |
| **219.** | **NFE2L1** | ENSG00000082641 |
| **220.** | **TGFB2** | ENSG00000092969 |
| **221.** | **GAB1** | ENSG00000109458 |
| **222.** | **AHSA2** | ENSG00000173209 |
| **#** | **Gene Name** | **Gene Ensembl id** |
| **223.** | **GTF2H3** | ENSG00000111358 |
| **224.** | **TP53BP1** | ENSG00000067369 |
| **225.** | **BCCIP** | ENSG00000107949 |
| **226.** | **REV3L** | ENSG00000009413 |
| **227.** | **MRE11A** | ENSG00000020922 |
| **228.** | **TOPORS** | ENSG00000197579 |
| **229.** | **CFH** | ENSG00000000971 |
| **230.** | **PARP1** | ENSG00000143799 |
| **231.** | **CCR1** | ENSG00000163823 |
| **232.** | **SOD2** | ENSG00000112096 |
| **233.** | **TP53I11** | ENSG00000175274 |
| **234.** | **ACTL6A** | ENSG00000136518 |
| **235.** | **PIK3CA** | ENSG00000121879 |
| **236.** | **PAPD7** | ENSG00000112941 |
| **237.** | **PLEKHA1** | ENSG00000107679 |
| **238.** | **LRRFIP1** | ENSG00000124831 |
| **239.** | **KIF22** | ENSG00000079616 |
| **240.** | **MALT1** | ENSG00000172175 |
| **241.** | **FOXO3** | ENSG00000118689 |
| **242.** | **DERL1** | ENSG00000136986 |
| **243.** | **BCL2A1** | ENSG00000140379 |
| **244.** | **USP7** | ENSG00000187555 |
| **245.** | **BAG6** | ENSG00000204463 |
| **246.** | **CDKN1A** | ENSG00000124762 |
| **247.** | **PKD2** | ENSG00000118762 |
| **248.** | **IRAK2** | ENSG00000134070 |
| **249.** | **CLSPN** | ENSG00000092853 |
| **250.** | **TNRC6A** | ENSG00000090905 |
| **251.** | **POLR3B** | ENSG00000013503 |
| **252.** | **MYBBP1A** | ENSG00000132382 |
| **253.** | **ATP2B4** | ENSG00000058668 |
| **254.** | **PTX3** | ENSG00000163661 |
| **255.** | **ZBTB1** | ENSG00000126804 |
| **256.** | **SUGT1** | ENSG00000165416 |
| **257.** | **DDX1** | ENSG00000079785 |
| **258.** | **PCBP2** | ENSG00000197111 |
| **259.** | **STAT1** | ENSG00000115415 |
| **260.** | **PRKDC** | ENSG00000253729 |
| **261.** | **SMAD7** | ENSG00000101665 |
| **262.** | **MEF2C** | ENSG00000081189 |
| **263.** | **UBE2W** | ENSG00000104343 |
| **264.** | **RHBDD1** | ENSG00000144468 |
| **265.** | **HERPUD2** | ENSG00000122557 |
| **266.** | **PLK1** | ENSG00000166851 |
| **267.** | **IFITM2** | ENSG00000185201 |
| **268.** | **ZBTB32** | ENSG00000011590 |
| **269.** | **PPIF** | ENSG00000108179 |
| **270.** | **ADD1** | ENSG00000087274 |
| **271.** | **JADE1** | ENSG00000077684 |
| **272.** | **ULK1** | ENSG00000177169 |
| **273.** | **MOAP1** | ENSG00000165943 |
| **274.** | **HSPA1B** | ENSG00000204388 |
| **275.** | **CHST1** | ENSG00000175264 |
| **276.** | **MX2** | ENSG00000183486 |
| **277.** | **FAM111A** | ENSG00000166801 |
| **278.** | **MAP2K4** | ENSG00000065559 |
| **#** | **Gene Name** | **Gene Ensembl id** |
| **279.** | **XRCC5** | ENSG00000079246 |
| **280.** | **VEGFA** | ENSG00000112715 |
| **281.** | **IRF7** | ENSG00000185507 |
| **282.** | **PTEN** | ENSG00000171862 |
| **283.** | **DTX3L** | ENSG00000163840 |
| **284.** | **DNAJB4** | ENSG00000162616 |
| **285.** | **SGK3** | ENSG00000104205 |
| **286.** | **ERRFI1** | ENSG00000116285 |
| **287.** | **IRAK1** | ENSG00000184216 |
| **288.** | **IRAK4** | ENSG00000198001 |
| **289.** | **SOX2** | ENSG00000181449 |
| **290.** | **MAPK1** | ENSG00000100030 |
| **291.** | **NIPBL** | ENSG00000164190 |
| **292.** | **MAP3K7** | ENSG00000135341 |
| **293.** | **RAD54L** | ENSG00000085999 |
| **294.** | **RNF168** | ENSG00000163961 |
| **295.** | **TLN1** | ENSG00000137076 |
| **296.** | **CD84** | ENSG00000066294 |
| **297.** | **ITGB2** | ENSG00000160255 |
| **298.** | **PRDX3** | ENSG00000165672 |
| **299.** | **CXCL10** | ENSG00000169245 |
| **300.** | **IL10** | ENSG00000136634 |
| **301.** | **ADCY9** | ENSG00000162104 |
| **302.** | **HSPE1** | ENSG00000115541 |
| **303.** | **SOD3** | ENSG00000109610 |
| **304.** | **HSPA1B** | ENSG00000212866 |
| **305.** | **TFF3** | ENSG00000160180 |
| **306.** | **RSAD2** | ENSG00000134321 |
| **307.** | **RHOB** | ENSG00000143878 |
| **308.** | **RAD9A** | ENSG00000172613 |
| **309.** | **JMY** | ENSG00000152409 |
| **310.** | **SETX** | ENSG00000107290 |
| **311.** | **HYOU1** | ENSG00000149428 |
| **312.** | **MEF2A** | ENSG00000068305 |
| **313.** | **SGK1** | ENSG00000118515 |
| **314.** | **MDM2** | ENSG00000135679 |
| **315.** | **PSMB5** | ENSG00000100804 |
| **316.** | **FANCI** | ENSG00000140525 |
| **317.** | **ABL1** | ENSG00000097007 |
| **318.** | **ERBB4** | ENSG00000178568 |
| **319.** | **BAZ1B** | ENSG00000009954 |
| **320.** | **SMG1** | ENSG00000157106 |
| **321.** | **BMPR2** | ENSG00000204217 |
| **322.** | **TMBIM6** | ENSG00000139644 |
| **323.** | **CLOCK** | ENSG00000134852 |
| **324.** | **MAP3K5** | ENSG00000197442 |
| **325.** | **SERPINA1** | ENSG00000197249 |
| **326.** | **TRIM26** | ENSG00000234127 |
| **327.** | **FNIP2** | ENSG00000052795 |
| **328.** | **TXNIP** | ENSG00000265972 |
| **329.** | **CCR6** | ENSG00000272980 |
| **330.** | **UHRF1** | ENSG00000276043 |
| **331.** | **CTDSP2** | ENSG00000175215 |
