# Supplementary material for: Recovery from 6-month spaceflight at the International Space Station: muscle-related stress into a proinflammatory setting
Source: FASEB J. 2019 Jan 8;33(4):5168–80. doi: 10.1096/fj.201801625R (PMC6436655; doi:10.1096/fj.201801625R)
Supplement: Supplementary file 6 [file fj.201801625R.st2.docx]

**Table S2.** List of validated genes which are targets of myo-miR-206.

| **#** | **Gene Name** | **Gene Ensembl id** |
| --- | --- | --- |
| **1.** | **EFNB2** | ENSG00000125266 |
| **2.** | **ESR1** | ENSG00000091831 |
| **3.** | **RPA1** | ENSG00000132383 |
| **4.** | **TWF1** | ENSG00000151239 |
| **5.** | **FAM57A** | ENSG00000167695 |
| **6.** | **ANP32E** | ENSG00000143401 |
| **7.** | **LIG1** | ENSG00000105486 |
| **8.** | **PICALM** | ENSG00000073921 |
| **9.** | **NETO2** | ENSG00000171208 |
| **10.** | **DTL** | ENSG00000143476 |
| **11.** | **TM4SF1** | ENSG00000169908 |
| **12.** | **MET** | ENSG00000105976 |
| **13.** | **SRSF9** | ENSG00000111786 |
| **14.** | **BLCAP** | ENSG00000166619 |
| **15.** | **PTMA** | ENSG00000187514 |
| **16.** | **Bdnf** | ENSMUSG00000048482 |
| **17.** | **RNF38** | ENSG00000137075 |
| **18.** | **NELFCD** | ENSG00000101158 |
| **19.** | **CDK4** | ENSG00000135446 |
| **20.** | **MGAT5** | ENSG00000152127 |
| **21.** | **UNC119B** | ENSG00000175970 |
| **22.** | **AP3S1** | ENSG00000177879 |
| **23.** | **MEA1** | ENSG00000124733 |
| **24.** | **CDC25C** | ENSG00000158402 |
| **25.** | **MSH5** | ENSG00000204410 |
| **26.** | **LRRC8B** | ENSG00000197147 |
| **27.** | **ADAR** | ENSG00000160710 |
| **28.** | **PLAGL2** | ENSG00000126003 |
| **29.** | **ZNF43** | ENSG00000198521 |
| **30.** | **CCND2** | ENSG00000118971 |
| **31.** | **TACC3** | ENSG00000013810 |
| **32.** | **GMFB** | ENSG00000197045 |
| **33.** | **BRIP1** | ENSG00000136492 |
| **34.** | **KIF2A** | ENSG00000068796 |
| **35.** | **EPB41L4B** | ENSG00000095203 |
| **36.** | **CDCA8** | ENSG00000134690 |
| **37.** | **HPS4** | ENSG00000100099 |
| **38.** | **INPP5F** | ENSG00000198825 |
| **39.** | **GMNN** | ENSG00000112312 |
| **40.** | **PTBP1** | ENSG00000011304 |
| **41.** | **MPZL1** | ENSG00000197965 |
| **42.** | **OSTF1** | ENSG00000134996 |
| **43.** | **LMNB1** | ENSG00000113368 |
| **44.** | **TNKS2** | ENSG00000107854 |
| **45.** | **BRCA2** | ENSG00000139618 |
| **46.** | **WEE1** | ENSG00000166483 |
| **#** | **Gene Name** | **Gene Ensembl id** |
| **47.** | **EGFR** | ENSG00000146648 |
| **48.** | **NOTCH2** | ENSG00000134250 |
| **49.** | **RNF138** | ENSG00000134758 |
| **50.** | **HNRNPU** | ENSG00000153187 |
| **51.** | **HMGN1** | ENSG00000205581 |
| **52.** | **FSTL1** | ENSG00000163430 |
| **53.** | **MCM7** | ENSG00000166508 |
| **54.** | **ASF1B** | ENSG00000105011 |
| **55.** | **DEK** | ENSG00000124795 |
| **56.** | **TFPI** | ENSG00000003436 |
| **57.** | **TDG** | ENSG00000139372 |
| **58.** | **IER5** | ENSG00000162783 |
| **59.** | **CHEK1** | ENSG00000149554 |
| **60.** | **POLR2K** | ENSG00000147669 |
| **61.** | **G6PD** | ENSG00000160211 |
| **62.** | **HDAC4** | ENSG00000068024 |
| **63.** | **SERP1** | ENSG00000120742 |
| **64.** | **TAGLN2** | ENSG00000158710 |
| **65.** | **BRCA1** | ENSG00000012048 |
| **66.** | **GOLGA7** | ENSG00000147533 |
| **67.** | **WDHD1** | ENSG00000198554 |
| **68.** | **SMC4** | ENSG00000113810 |
| **69.** | **SLC5A3** | ENSG00000198743 |
| **70.** | **PIR** | ENSG00000087842 |
| **71.** | **CRTAP** | ENSG00000170275 |
| **72.** | **C2CD5** | ENSG00000111731 |
| **73.** | **GPD2** | ENSG00000115159 |
| **74.** | **ZNF264** | ENSG00000083844 |
| **75.** | **POGK** | ENSG00000143157 |
| **76.** | **AP1S1** | ENSG00000106367 |
| **77.** | **PKMYT1** | ENSG00000127564 |
| **78.** | **IFT52** | ENSG00000101052 |
| **79.** | **CENPF** | ENSG00000117724 |
| **80.** | **MTX1** | ENSG00000173171 |
| **81.** | **ACOT7** | ENSG00000097021 |
| **82.** | **CBX6** | ENSG00000183741 |
| **83.** | **MMD** | ENSG00000108960 |
| **84.** | **RBBP8** | ENSG00000101773 |
| **85.** | **TMCC1** | ENSG00000172765 |
| **86.** | **NXT2** | ENSG00000101888 |
| **87.** | **TSPAN4** | ENSG00000214063 |
| **88.** | **SNX2** | ENSG00000205302 |
| **89.** | **ATP6V1A** | ENSG00000114573 |
| **90.** | **NUP50** | ENSG00000093000 |
| **91.** | **CDC7** | ENSG00000097046 |
| **92.** | **DHX15** | ENSG00000109606 |
| **#** | **Gene Name** | **Gene Ensembl id** |
| **93.** | **BUB1B** | ENSG00000156970 |
| **94.** | **FAIM** | ENSG00000158234 |
| **95.** | **TOPBP1** | ENSG00000163781 |
| **96.** | **MCF2L-AS1** | ENSG00000235280 |
| **97.** | **GNPTAB** | ENSG00000111670 |
| **98.** | **MKI67** | ENSG00000148773 |
| **99.** | **POM121** | ENSG00000196313 |
| **100.** | **NOTCH3** | ENSG00000074181 |
| **101.** | **CHSY1** | ENSG00000131873 |
| **102.** | **S100PBP** | ENSG00000116497 |
| **103.** | **POLQ** | ENSG00000051341 |
| **104.** | **H3F3B** | ENSG00000132475 |
| **105.** | **TAC1** | ENSG00000006128 |
| **106.** | **PPP2R3B** | ENSG00000167393 |
| **107.** | **FUBP1** | ENSG00000162613 |
| **108.** | **TDP1** | ENSG00000042088 |
| **109.** | **LASP1** | ENSG00000002834 |
| **110.** | **FN1** | ENSG00000115414 |
| **111.** | **DIMT1** | ENSG00000086189 |
| **112.** | **GAS2L1** | ENSG00000185340 |
| **113.** | **GJA1** | ENSG00000152661 |
| **114.** | **TMEM30A** | ENSG00000112697 |
| **115.** | **HHLA3** | ENSG00000197568 |
| **#** | **Gene Name** | **Gene Ensembl id** |
| **116.** | **CAD** | ENSG00000084774 |
| **117.** | **Tppp** | ENSMUSG00000021573 |
| **118.** | **DCLRE1B** | ENSG00000118655 |
| **119.** | **CDC42SE1** | ENSG00000197622 |
| **120.** | **UTRN** | ENSG00000152818 |
| **121.** | **OAT** | ENSG00000065154 |
| **122.** | **ITPR3** | ENSG00000096433 |
| **123.** | **ARHGEF18** | ENSG00000104880 |
| **124.** | **RFWD3** | ENSG00000168411 |
| **125.** | **CENPU** | ENSG00000151725 |
| **126.** | **PLK4** | ENSG00000142731 |
| **127.** | **CAP1** | ENSG00000131236 |
| **128.** | **PPP2R1B** | ENSG00000137713 |
| **129.** | **RFC3** | ENSG00000133119 |
| **130.** | **ARF4** | ENSG00000168374 |
| **131.** | **MYBL2** | ENSG00000101057 |
| **132.** | **ABCC6** | ENSG00000091262 |
| **133.** | **MDC1** | ENSG00000137337 |
| **134.** | **EXO1** | ENSG00000174371 |
| **135.** | **PGD** | ENSG00000142657 |
| **136.** | **PAX3** | ENSG00000135903 |
| **137.** | **SYNCRIP** | ENSG00000135316 |
