# Supplementary material for: Recovery from 6-month spaceflight at the International Space Station: muscle-related stress into a proinflammatory setting
Source: FASEB J. 2019 Jan 8;33(4):5168–80. doi: 10.1096/fj.201801625R (PMC6436655; doi:10.1096/fj.201801625R)
Supplement: Supplementary file 7 [file fj.201801625R.sd1.docx]

**Supplemental figure legends**

**Figure S1. C-myo-miR-133a-3p and c-miR-122-5p**

**A and C.** Measurements of circulating myo-miR-133a-3p and c-miR-122-5p are respectively reported in dependence of time series analysis in both crewmembers. Circle represents pre-flight, triangle is (R+1 day) landing time and square denotes (R+15 days) recovery time. **B and D.** C-myo-miRs-133a-3p and c-miR-122-5p values of crewmembers are compared with age-matched control distribution (19 measurements). Blue color: crewmember A; Red color crewmember B.

**Figure S2. C-miR-145-5p and c-miR-363-3p**

**A and C.** Measurements of circulating miR-145-5p and c-miR-363-3p are respectively reported in dependence of time series analysis in both crewmembers. For explanation of symbols, see Figure S1. **B and D.** C-miRs-145-5p and c-miR-363-3p values of crewmembers are compared with age-matched control distribution (19 measurements). Blue color: crewmember A; Red color crewmember B.

**Figure S3. TGF-β1 and c-mtDNA**

**A and C.** Measurements of circulating TGF-β1 and c-mt-DNA are reported in dependence of time series analysis in both crewmembers. For explanation of symbols, see Figure S1. **B and D.** TGF-β1 and c-mt-DNA values of crewmembers are compared with age-matched control distribution (19 measurements). Blue color: crewmember A; Red color crewmember B.

**Figure S4. Proteomic data**

A representative example of HSPB1 analysis with MALDI-ToF PMF and electrospray ionization-MS/MS is reported as both spectrum (upper panel) and sequence analysis (lower panel), respectively.
